# Pathogenic waterborne free-living amoebae: An update from selected Southeast Asian countries

**DOI:** 10.1371/journal.pone.0169448

**Published:** 2017-02-17

**Authors:** Mohamad Azlan Abdul Majid, Tooba Mahboob, Brandon G. J. Mong, Narong Jaturas, Reena Leeba Richard, Tan Tian-Chye, Anusorn Phimphila, Panomphanh Mahaphonh, Kyaw Nyein Aye, Wai Lynn Aung, Joon Chuah, Alan D. Ziegler, Atipat Yasiri, Nongyao Sawangjaroen, Yvonne A. L. Lim, Veeranoot Nissapatorn

**Affiliations:** 1 Department of Parasitology (Southeast Asia Water Team), Faculty of Medicine, University of Malaya, Kuala Lumpur, Malaysia; 2 Department of Medical Laboratory, Faculty of Medical Technology, University of Health Sciences, Vientiane, Laos PDR; 3 Ecological Laboratory, Advancing Life and Regenerating Motherland (ALARM), Yangon, Myanmar; 4 Institute of Water Policy, National University of Singapore, Singapore, Singapore; 5 Department of Geography, National University of Singapore, Singapore, Singapore; 6 Chulabhorn International College of Medicine, Thammasat University, Pathum Thani, Thailand; 7 Department of Microbiology, Faculty of Science, Prince of Songkhla University, Hat-Yai, Thailand; National Cheng Kung University, TAIWAN

## Abstract

Data on the distribution of free-living amoebae is still lacking especially in Southeast Asian region. The aquatic environment revealed a high occurrence of free-living amoebae (FLA) due to its suitable condition and availability of food source, which subsequently causes infection to humans. A total of 94 water samples consisted of both treated and untreated from Laos (31), Myanmar (42), and Singapore (21) were investigated for the presence of pathogenic FLA. Each water sample was filtered and cultured onto non-nutrient agar seeded with live suspension of *Escherichia coli* and incubated at room temperature. Morphological identification was conducted for both trophozoites and cysts via microscopic stains (Giemsa and immunofluorescence). The presence of *Naegleria*-like structures was the most frequently encountered in both treated and untreated water samples, followed by *Acanthamoeba*-like and *Vermamoeba*-like features. To identify the pathogenic isolates, species-specific primer sets were applied for molecular identification of *Acanthamoeba*, *Naegleria*, and *Vermamoeba*. The pathogenic species of *Acanthamoeba lenticulata* and *A*. *triangularis* were detected from untreated water samples, while *Vermamoeba vermiformis* was found in both treated and untreated water samples. Our results suggested that poor water quality as well as inadequate maintenance and treatment might be the cause of this alarming problem since chlorine disinfection is ineffective in eradicating these amoebas in treated water samples. Regular monitoring and examination of water qualities are necessary in order to control the growth, hence, further preventing the widespread of FLA infections among the public.

## Introduction

Free-living amoebae (FLA) belonging to the genera *Acanthamoeba*, *Balamuthia*, *Naegleria*, *Sappinia*, and *Vermamoeba* (= *Hartmannella*) are potentially pathogenic to humans [1,2]. They are also known as amphizoic amoeba due to the ability to exist within a host or in the environment as ‘free-living’. They are able to survive and proliferate in the environment independently and can be found in various natural and man-made aquatic environments, both fresh and marine, including lakes, ponds, swimming pools, and even treated water supplies [3–5]. The amoeba cysts are highly resistant to harsh conditions due to the process of encystment [6,7].

Many species under the genus of *Acanthamoeba* are reported to be the causative agent of keratitis in healthy individuals, often among contact lens wearers. In opportunistic infections, *Acanthamoeba* species can cause pneumonitis, fatal granulomatous encephalitis and skin infections [8]. To date, 20 different genotypes (T1-T20) of *Acanthamoeba* have been described [9]. Meanwhile, *Naegleria* is the only FLA that has the advantage of exhibiting a flagellate stage to ease its movement by swimming in water. Moreover, more than 40 species of *Naegleria* have been identified, only *N*. *fowleri* is found to be the causative agent of primary amoebic meningoencephalitis (PAM), a lethal brain infection [10]. Approximately, 440 of PAM cases have been reported worldwide until the year 2008 [11], with exposure of healthy individuals to warm (water temperatures of 25 to 44°C), untreated or poorly disinfected water systems. In addition, *Vermamoeba* (= *Hartmannella*) *vermiformis* seems to be the potential causative agent of human keratitis [12] and it can serves as a host to pathogenic bacteria, *Legionella pneumophila* [13,14].

In Southeast Asia, the occurrence of FLA were reported in Malaysia [15,16], Thailand [17], Vietnam [18] and the Philippines [19]. Although the reported cases are extremely rare, the diagnosis of FLA must never be overlooked as it is able to cause serious and fatal diseases. Keratitis infection caused by *Acanthamoeba* species was reported among contact lens wearers in Malaysia [20] and Singapore [21]. Meanwhile, *N*. *fowleri*, was reported to be the etiological agent for 12 cases of PAM in Thailand [22,23]. In addition, the first case of *Balamuthia mandrillaris* causing meningoencephalitis was reported from a Thai male patient after falling into a swamp [24].

The knowledge of pathogenic FLA emergence has gained much interest throughout the world due to its possible health implications. However, inadequate studies of FLA attributed to the lack of prevalence information across the Southeast Asian region. Therefore, we undertook an investigation to detect the occurrence and distribution of the FLA, together with the identification based on culture, staining, and molecular assay of each isolate. This information should be useful for early detection of potential infestation of pathogenic FLA in various water sources and further evaluating the risks of human contact with FLA. In fact, humans can be exposed to FLA via accidental splash/squirt of contaminated water to the face or through an open wound, hence, necessitating this study in bridging the gaps of awareness on FLA.

## Materials and methods

### Ethics statement/Permission approval

This study was carried out in three selected Southeast Asian countries namely; Lao PDR, Myanmar and Singapore during 2013 to 2015. Invitation letters were approved from the relevant institutions namely University of Health Sciences, Vientiane, Lao PDR, Advancing Life and Regenerating Motherland (ALARM), Yangon, Myanmar, and National University of Singapore, Singapore. The collaborators from each country had been consulted at the time of water samples collection.

### Sample collection

**Fig 1:**
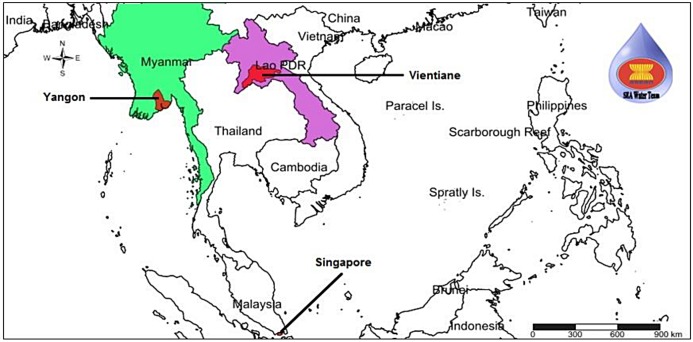
Sampling locations in Laos, Myanmar, and Singapore.

**Fig 2:**
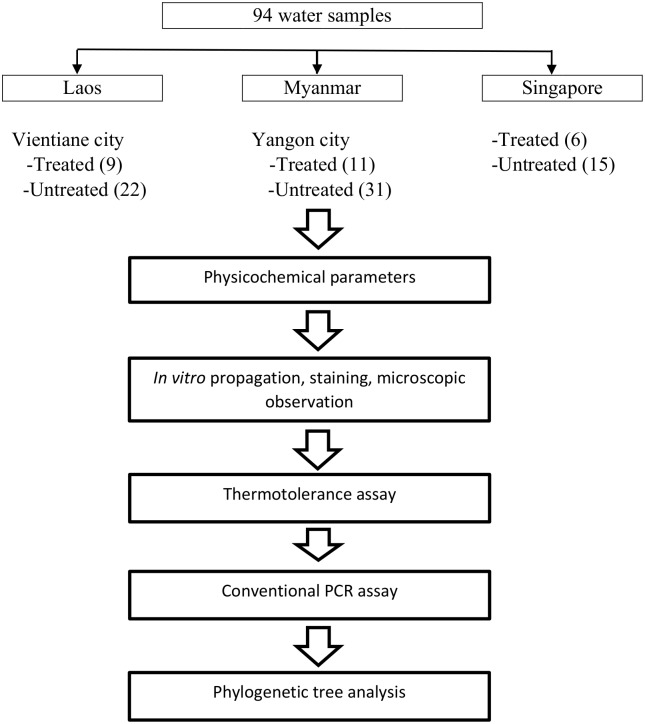
Flowchart of the overall water analysis.

A total of 94 samples of either treated or untreated water were collected at various locations in Vientiane, Laos (31), Yangon, Myanmar (42) and Singapore (21) (Figs 1 and 2). From each sampling point, four samples were collected in 50 mL sterile centrifuge tubes. The tubes were submerged beneath the surface of untreated water, while treated water obtained from pipes was allowed to flow into the tubes. All of the water samples were transported to the laboratory and processed within 4 hours (hrs) after sampling.

### Physicochemical analysis of water quality

Physical parameters (YSI 556 Multiprobe System, USA) of the water samples such as turbidity (NTU), temperature (°C), total dissolved solids (mg/L), salinity (PSU), and dissolved oxygen (mg/L) content were measured at the sampling sites. Baseline chemical parameters (e.g., ammonia, chlorine, nitrite, nitrate, and fluoride) were measured *in situ* using colorimeter (Hach DR/890 Portable Colorimeter, USA) and recorded as mean values of the overall sites from each country.

### Isolation of free-living amoebae

Prior to cultivation, the samples were centrifuged at 2000 rpm for 15 minutes (mins). One or two drops of sediment samples were spread onto the non-nutrient agar plates coated with a layer of *Escherichia coli* (NNA-*E*. *coli*). The plates were then incubated at room temperature (25–28°C) for 1 week.

### Morphological observation and identification

The first line plates were observed daily (up to 7 days) using an inverted microscope. The morphological appearances of the amoebic isolates were identified according to Page’s taxonomy keys [25,26]. The trophozoite and cyst stages of the amoebae were flushed from the culture plates and were subjected for direct screening, Giemsa and FITC-DAPI staining. For each microscopy observation, 25 μL of amoebic isolates were placed on a glass slide. Both direct screening and Giemsa staining were examined using light microscope (Olympus BX51, Japan). Giemsa stain was prepared by submerging the methanol-fixed slide into a mixture of 20 mL of buffered distilled water (pH 7.1), followed by 40 drops of Giemsa stain. For FITC-DAPI staining, each sample was stained with 50 μL of FITC-MAb of *Giardia*/*Cryptosporidium* (CeLLabs, Brookvale, Australia) and incubated in a humidified chamber for 37°C for 30 mins, followed by washing step using PBS solution (pH 7.2). The sample was further stained with 50 μL of DAPI solution (Louis, Missouri, USA) for 2 mins before the addition of 50 μL of distilled water for 1 min. Finally, a 20 μL aliquot of mounting medium was placed onto the slide, covered with coverslip, and left air-dried prior to examination under epifluorescence microscope (Olympus BX51, Tokyo, Japan).

### Thermotolerance assay

Thermotolerance assay was carried out for the selected FLA isolates (*V*. *vermiformis* and *Acanthamoeba* spp.) that had been confirmed through microscopy examination. Sub-culturing was performed as described above to obtain axenic culture of the amoeba. The samples were introduced to certain temperatures (34°C, 37°C, 40°C and 45°C) for a period of 24 hrs, respectively [27]. The isolates were then observed microscopically to confirm its viability.

### DNA extraction

The trophozoites stage of each amoebic isolate were harvested from the plates with 5 mL of cold Page’s Amoeba Saline (PAS) and transferred into 1.5 mL tubes. DNA was further extracted using a commercial QIAamp DNA Blood Mini Kit (Qiagen, Hilden, France) following the manufacturer’s procedures and stored at -20°C until further analysis.

### PCR amplification

For *Acanthamoeba* species, amplification was carried out by targeting the 18S region, with a PCR mix containing 10X DNA polymerase (Thermo Scientific, Lithuania, USA), 25 mM of magnesium chloride (MgCl_2_) (Thermo Scientific, Lithuania, USA), 10 mM of deoxynucleotide triphosphate (dNTP) mix (Thermo Scientific, Lithuania, USA) and 1 U/μL of Taq DNA polymerase (Thermo Scientific, Lithuania, USA) with 200 nmoles of each primer: JDP1 (5`-GGCCCAGATCGTTACCGTGAA–3`) and JDP2 (5`- TCTCACAAGCTGCTAGGGAGTCA– 3`) [28], with 5 ng of DNA templates. The reaction was performed at 94°C for 5 mins, followed by 40 cycles at 94°C for 1 min, 60°C for 1 min, 72°C for 1 min, and an extension at 72°C for 5 mins [28].

*N*. *fowleri* was detected by amplification of the ITS region (ITS1, 5.8S, and ITS2) using species-specific primers: the forward primer NFITSFW (5’-TGAAAACCTTTTTTCCATTTACA-3’) and the reverse primer NFITSRV (5’-AATAAAAGATTGACCATTTGAAA-3’) [29]. Amplifications were performed in a PCR mix containing 10X DNA polymerase buffer (Thermo Scientific, Lithuania, USA), 25 mM of magnesium chloride (MgCl_2_) (Thermo Scientific, Lithuania, USA), 10 mM of deoxynucleotide triphosphate (dNTP) mix (Thermo Scientific, Lithuania), 200 nmoles of each primers, and 1 U/μL of Taq DNA polymerase (Thermo Scientific, Lithuania, USA), with 5 ng of DNA templates. The PCR temperature profiles consisted of 94°C for 6 mins, followed by 30 cycles at 94°C for 1 min, 55°C for 1.5 mins, 72°C for 2 mins and the elongation step at 72°C for 10 mins [29].

The PCR reaction of *H*. *vermiformis* was performed in a 20 μL of Pre-mix (Thermo Scientific, Lithuania, USA) with 200 nmoles primers: the forward primer NA1 (5’-GCT CCA ATA GCG TAT ATT AA-3’) and the reverse primer NA2 (5’-AGA AAG AGC TAT CAA TCT GT-3’) [30] with 5 ng of DNA templates. The PCR cycling condition included the denaturation at 94°C for 1 min, followed by 35 repetition cycles at 94°C for 35 seconds, 56°C for 45 seconds, 72°C for 1 min, and the final elongation at 72°C for 5 mins [30].

Amplifications were conducted in a thermal cycler (BioRad, Hercules, USA) with the amount of 50–100 ng DNA templates in a final volume of 25 μL. PCR amplicon was run in a 1.5% agarose gel in Tris-Acetate-EDTA (TAE) buffer (Thermo Scientific, Lithuania, USA) at 100 V for 45 mins. A 100-bp DNA ladder was used to compare the expected amplicon sizes in the gel. Post-staining method was carried out using ethidium bromide (Amresco, Ohio, USA) and visualized under UV-transilluminator.

### Nucleotide sequencing and phylogenetic analysis

Positive samples of PCR were purified using QIAamp PCR purification kit (Qiagen, Hilden, France) and sent for sequencing with the respective forward and reverse primers. A homology search was conducted for each sequence results with the FLA species deposited in the GenBank using Basic Local Alignment Search Tool (BLAST) software provided by the National Centre for Biotechnology Information (NCBI). The similarity between the species were compared by performing maximum-likelihood into phylogenetic tree using MEGA version 6 software, followed by Kimura 2-parameter algorithm with bootstrap analysis of 1000 replicates. In addition, *V*. *vermiformis* from ice-cube sample in our study was compared to a similar isolate (i.e. SS1 and SS2 strains) discovered from snow sample in Spain [31].

## Results

Table 1 summarizes the physicochemical analysis of water quality parameters, presented as calculated means ± standard deviation (SD), with 95% confidence intervals (CIs). Overall results showed untreated water samples from Myanmar had the highest readings of turbidity (65.21±14.85 NTU; CI: 29.53), TDS (121.30±94.20 mg/L; CI: 35.90), chlorine (0.28±0.39 mg/L; CI: 0.18). The presence of *Acanthamoeba* and *Vermamoeba* were detected in water samples with high TDS and turbidity. In Myanmar, *Vermamoeba* was the only pathogenic FLA found in treated water samples with high nitrate concentration (2.13±6.03 mg/L; CI: 3.34). In Laos, *Acanthamoeba* was detected in untreated water samples with high level of nitrite (0.38±0.20 mg/L; CI: 0.13) and *Vermamoeba* was also detected in treated water samples with high level of ammonia (2.05±5.36 mg/L; CI: 3.10).

**Table 1:** The analysis of physicochemical water quality variables from treated and untreated samples in Laos, Myanmar, and Singapore.

Country	Type of water	Mean, standard deviation (SD), and confidence intervals (CI)	Physicochemical water quality variables							
			Physical				Chemical			
			Turbidity	TDS^d^	Salinity	DO^e^	Chlorine	Nitrate	Nitrite	Ammonia
			(NTU)^c^	(mg/l)	(PSU)	(mg/l)	(mg/l)	(mg/l)	(mg/l)	(mg/l)
Laos	Treated^a^	Mean	4.05	50.75	0.05	3.11	0.19	0.19	0.10	2.05^g^
		SD	3.71	43.30	0.04	3.25	0.32	0.14	0.15	5.36
		CI (95%)	2.70	63.70	0.03	2.82	0.26	0.15	0.11	3.10
	Untreated^b^	Mean	20.55^i^	57.88^i^	0.05	2.65	0.16	0.32	0.38^f^	0.55
		SD	15.75	68.45	0.02	2.52	0.15	0.41	0.20	0.51
		CI (95%)	8.06	28.62	0.15	0.75	0.05	0.14	0.13	0.17
Myanmar	Treated^a^	Mean	1.34	85.30	0.25	5.42	0.22	2.13^h^	0.05	0.74
		SD	1.99	117.80	0.54	6.81	0.75	6.03	0.03	1.90
		CI (95%)	0.85	50.75	0.39	2.46	0.31	3.34	0.04	1.42
	Untreated^b^	Mean	65.21^i^	121.30^i^	1.39	2.55	0.28	1.29	0.35	0.19
		SD	14.85	94.20	3.88	1.85	0.39	0.93	1.14	0.25
		CI (95%)	29.53	35.90	0.95	0.36	0.18	0.15	0.27	0.08
Singapore	Treated^a^	Mean	1.01	15.13	0.03	0.52	0.25	0.20	0.01	0.13
		SD	0.54	25.15	0.01	0.92	0.18	0.18	0.01	0.15
		CI (95%)	0.55	16.11	0.02	0.54	0.25	0.14	0.00	0.16
	Untreated^b^	Mean	5.48	30.25	0.05	1.64	0.22	0.19	0.20	0.42
		SD	2.35	21.95	0.03	1.82	0.45	0.24	0.32	0.31
		CI (95%)	1.88	48.87	0.04	0.83	0.20	0.17	0.13	0.18

^a^Treated water includes drinking water, water dispenser, mineral water, tap water, and swimming pools

^b^Untreated water includes rain water, springs, wells, recreational lake, rivers, waterfalls, canals/channels and effluent water

^c^NTU is nephelometric turbidity unit

^d^TDS is total dissolved solids

^e^DO is dissolved oxygen

^f^*Acanthamoeba* was detected in untreated water samples with high level of nitrite

^h^*Vermamoeba* was detected in treated water samples with high concentration of nitrate

^i^*Acanthamoeba* and *Vermamoeba* were detected in untreated water samples with high level of TDS and turbidity

**Fig 3:**
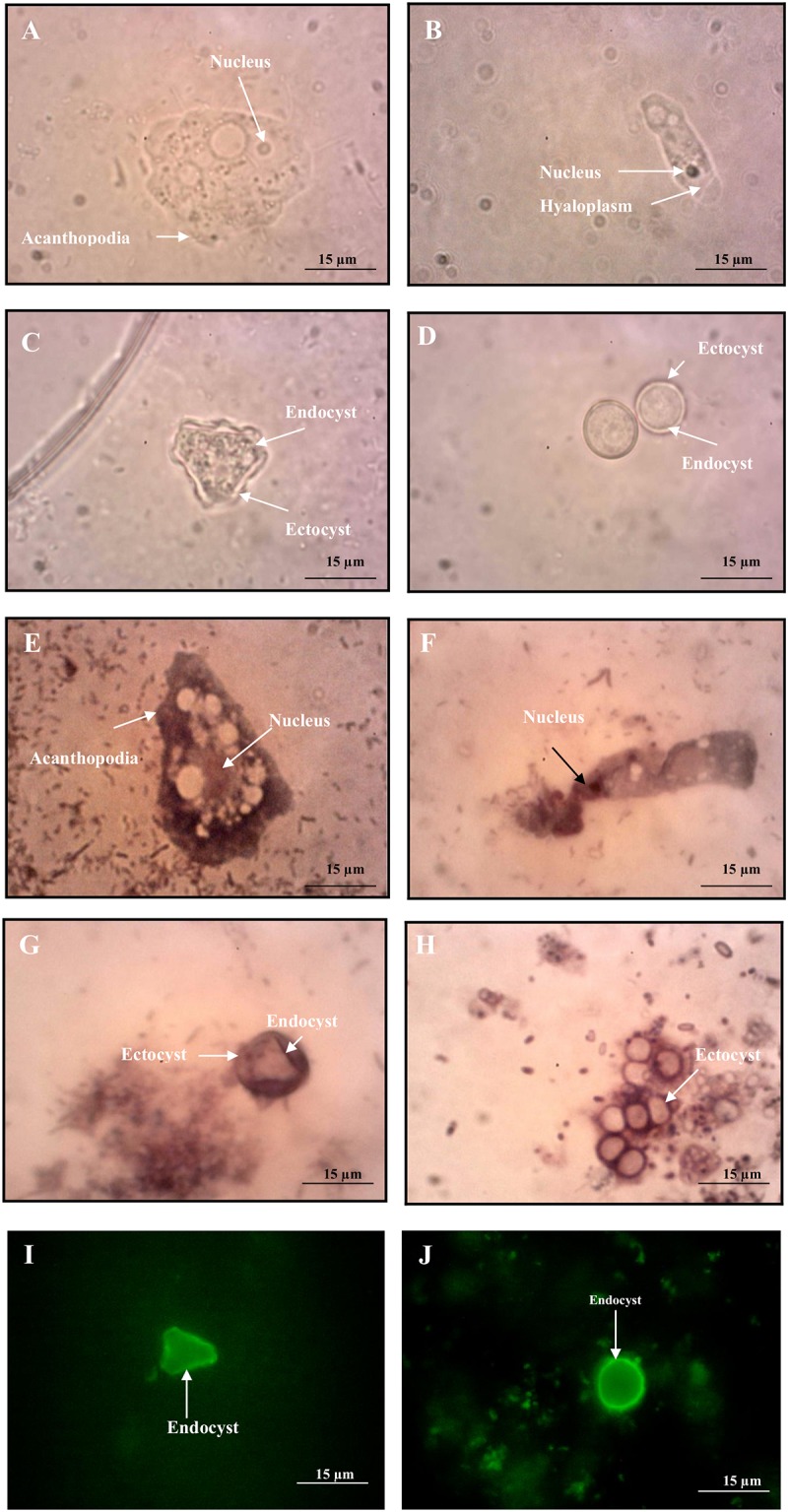
Morphological observations of trophozoite and cyst. (A,E) *Acanthamoeba* trophozoite, (C,G) *Acanthamoeba* cyst, (B,F) *Hartmannella* trophozoite, (D,H) *Hartmannella* cyst under light microscope (LiM) and (I) *Acanthamoeba* cyst, (J) *Hartmannella* cyst under epifluorescencce microscope (EpM). **(A**, LiM X 400; **E**, LiM X 400) *Acanthamoeba* trophozoites showing typical eruptive pseudopodia/lobopodia with no stain (A) and Giemsa stain (E) **(C**, LiM X 400; **G**, LiM X 400) A single *Acanthamoeba* cyst showing smooth ectocyst and endocyst with no stain (C) and Giemsa stain (G) **(B**, LiM X 400; **F**, LiM X 400) *Vermamoeba* trophozoite with no stain (B) and Giemsa stain (F) **(D**, LiM X 400; **H**, LiM X 400) Rounded form of *Vermamoeba* cyst with no stain (D) and Giemsa stain (H) **(I**, EpM X 400; **J**, EpM X 400) Triangular shape of *Acanthamoeba* cyst (I) and rounded form of *Vermamoeba* cyst (J) with immunofluorescence stain.

Initially, positive amoebic-like cells exist as mixed isolates with other organisms. The trophozoite and cyst stages were normally seen after 2–3 days and at least 5–6 days of cultivation, respectively. Both stages were photographed in Fig 3. All isolates were grown at room temperature. The morphology of all isolates can be clearly differentiated based on its motile stage of trophozoite/flagella. The *Acanthamoeba*-like cell exhibited irregular shape with distinct projection of pseudopodia/acanthopodia in multidirectional movement. The *Vermamoeba*-like trophozoite showed a predominant cylindrical form with monopodia and moving in one direction. Meanwhile, for *Naegleria*-like cell, the flagellate stage was observed on the watery surface of the medium agar (not shown).

Two staining methods (i.e. Giemsa and immunofluorescence) were used for the morphological identification of pathogenic amoebae and were compared with the non-stained slides as a control. In control slides, the nucleus and cytoplasm of the trophozoite were clearly distinguished, whereas two distinct layers of the cyst were observed for both *Acanthamoeba* and *Vermamoeba* isolates (Fig 3A–3D). For Giemsa, the trophozoite and cyst of the parasites were stained purple and showed a good contrast with the background. The eruptive pseudopodia of *Acanthamoeba* and the cylindrical form of *Vermamoeba* trophozoites were seen but unclear colour contrast between nucleus and cytoplasm was encountered (Fig 3E and 3F). Nevertheless, both outer and inner layers of the cysts were successfully observed as dark purple in colour (Fig 3G and 3H). The USEPA method 1623 was implemented to stain the amoebic cysts, similarly to *Cryptosporidium* and *Giardia* (oo)cysts. The inner layer was stained green, whereas the outer layer was appeared unstained against the dark background (Fig 3I and 3J). From the observation of thermotolerance assay, the trophozoites did not transformed into cyst stage for all of the tested temperatures.

The *Vermamoeba*-like trophozoite was detected in 1 of the 11 treated samples (9.1%) and 2 out of 31 untreated (6.5%) from Myanmar. In Laos, it was detected in 1 out of 9 treated samples (11.1%). *Vermamoeba sp*. was not detected in the samples collected in Singapore (Table 2). *Naegleria*-like flagella was the most frequently encountered in both treated and untreated water samples (Table 2). From Myanmar samples, it was found in all 11 treated water samples (100%) and 28 out of 31 untreated samples (90.3%). Samples from Laos showed the presence of flagella in 7 out of 9 treated samples (77.8%) and 18 of the 22 untreated samples (81.8%). Meanwhile, flagella were observed in 8 out of 15 untreated samples (53.3%) from Singapore. The *Acanthamoeba* isolate was observed only in untreated water samples (Table 2); 3 out of 31 samples (9.7%) from Myanmar, 1 out of 22 samples (4.5%) from Laos, and 1 of the 15 samples (6.7%) from Singapore.

**Table 2:** The occurrence of pathogenic FLA via microscopic examination and PCR in Laos, Myanmar, and Singapore.

Country of origin	Type of water	No. of samples	Free-living amoeba (FLA)					
			*Vermamoeba* sp.		*Naegleria* sp.		*Acanthamoeba* sp.	
			M (n)	PCR (n)	M (n)	PCR (n)	M (n)	PCR (n)
Laos	Treated^a^	9	1	*V*. *vermiformis* (1)	7	ND^c^	ND^c^	ND^c^
	Untreated^b^	22	ND^c^	ND^c^	18	ND^c^	1	*A*. *lenticulata* (1)
Myanmar	Treated^a^	11	2	*V*. *vermiformis* (2)	11	ND^c^	ND^c^	ND^c^
	Untreated^b^	31	2	*V*. *vermiformis* (2)	28	ND^c^	3	*A*. *lencticulata* (2); *A*. *triangularis* (1)
Singapore	Treated^a^	6	ND^c^	ND^c^	ND^c^	ND^c^	ND^c^	ND^c^
	Untreated^b^	15	ND^c^	ND^c^	8	ND^c^	1	ND^c^
Total		94						

^a^Treated water includes drinking water, water dispenser, mineral water, tap water, and swimming pools

^b^Untreated water includes rain water, springs, wells, recreational lake, rivers, waterfalls, canals/channels and effluent water

^c^ND = not detected; M = Microscopy; n = Number of samples; PCR = Polymerase chain reaction; V = *Vermamoeba*; A = *Acanthamoeba*

**Fig 4:**
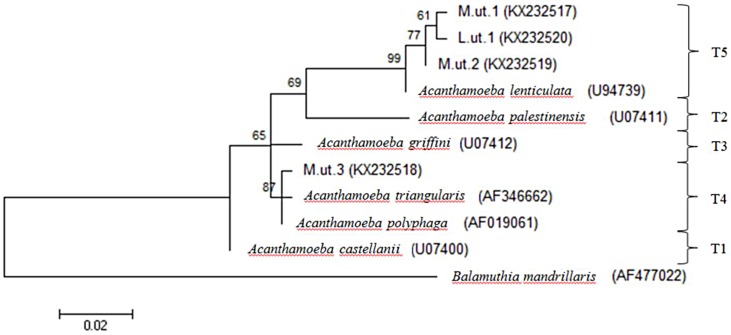
Phylogenetic analysis of pathogenic free-living *Acanthamoeba* spp. isolated from various types of water sources in selected Southeast Asian countries.

**Fig 5:**
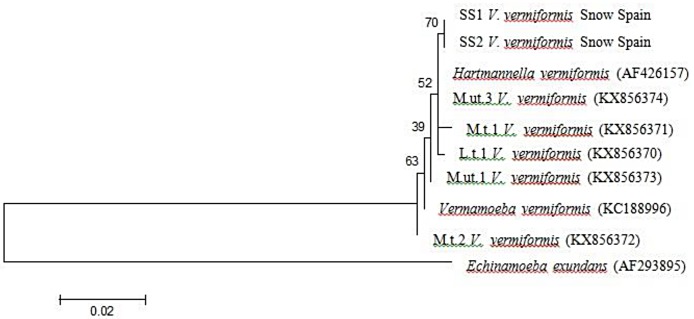
Phylogenetic analysis of pathogenic free-living *Vermamoeba vermiformis* isolated from various types of water sources in selected Southeast Asian countries.

After PCR amplification, none of the 94 isolates showed amplicon bands of *N*. *fowleri* using the ITS species-specific primer. For *Acanthamoeba* typing, the DF3 region of 18S rRNA was amplified, producing a 450bp amplicon. *A*. *triangularis* was detected from one isolate (KX232518) from Myanmar and formed a clade with both *A*. *triangularis* (AF346662) and *A*. *polyphaga* (AF019061) in T4 group. Another two isolates (KX232517, KX232519) from Myanmar and one isolate (KX232520) from Laos were identified as *A*. *lenticulata*. They were grouped as T5 and formed a clade with *A*. *lenticulata* (U94739). *Balamuthia mandrillaris* (AF477022) that doesn’t contain DF3 region showed distantly related with the *Acanthamoeba* groups (Table 3, Fig 4). A 800 bp PCR product was obtained for all of the *Vermamoeba* isolates (KX856370, KX856371, KX856372, KX856373, KX856374) and sequence analysis revealed that the amoebae had a high homology of 99% to *V*. *vermiformis* (KC188996). All other *V*. *vermiformis* strains, including 5 environmental sequences from present study, clearly showed a clade with each other under the group of Vermamoebidae. At the family level, *E*. *exundans* formed a distinct group of Echinamoebidae against the Vermamoebidae, within the order of Echinamoebida (Table 3, Fig 5). *V*. *vermiformis* pairwise distance between ice cube isolate and snow strain from Spain showed 0.5% intraspecific variation. BLAST results revealed the similarity at 99% and 98% of ice cube isolate-SS1 and ice cube isolate-SS2 strains, respectively (data not shown).

**Table 3:** Pathogenic FLA isolated from selected Southeast Asian countries.

Pathogenic FLA	Code	Accession number	Country of origin	Source of water
*A*. *lenticulata*	M.ut.1	KX232517	Myanmar	Well water
*A*. *lenticulata*	M.ut.2	KX232519	Myanmar	Recreational lake
*A*. *lenticulata*	L.ut.1	KX232520	Laos	Mekong up 2
*A*. *triangularis*	M.ut.3	KX232518	Myanmar	Fish pond 2
*V*. *vermiformis*	M.t.1	KX856371	Myanmar	Ice cube
*V*. *vermiformis*	M.t.2	KX856372	Myanmar	Swimming pool 2
*V*. *vermiformis*	M.ut.1	KX856373	Myanmar	Well water
*V*. *vermiformis*	M.ut.3	KX856374	Myanmar	Fish pond 2
*V*. *vermiformis*	L.t.1	KX856370	Laos	Swimming pool adult

V = *Vermamoeba*; A = *Acanthamoeba*; FLA = Free-living amoeba

## Discussion and conclusions

Investigations of free-living amoebae have been dramatically increased because of their important role within the ecosystems and their ability to cause serious infections among humans. With the ever growing number of FLA cases, various studies had been carried out throughout the world, especially from environmental waters, in assisting to decrease the potential source of contamination. Several studies conducted in Peninsular Malaysia [15,16] and the Philippines [19] showed occurrence of FLA in environmental water samples even they were proven to be non-pathogenic using PCR assay. To our knowledge, the present study is the first comprehensive report of the occurrence of potentially pathogenic FLA in both treated and untreated water samples from selected Southeast Asian countries.

Here, we investigated the occurrence of FLA both from raw and treated water sources from three (3) selected Southeast Asian countries and demonstrated the distribution of the potential pathogenic species by both microscopy and molecular approaches. Following PCR and sequencing, only 2 families of potentially pathogenic amoeba belongs to the genera of *Acanthamoeba* (Acanthamoebidae) and *Vermamoeba* (Vermamoebidae) were found. Microscopically, Myanmar reported the highest occurrence of FLA (92%, 39/42) followed by Laos (87%, 27/31), and Singapore (43%, 9/21). The rate of FLA in Laos and Myanmar were surprisingly found higher in comparison to a previous study conducted in this region such as Thailand (45.2%, 43/95) [17], though these countries share much similarity in geographical distribution. Moreover, this figure is remarkably higher than other countries namely; Japan (49.5%, 47/95) [17], USA (43.4%, 143/330) and Iran (35%, 42/120), but lower than Bulgaria (93.9%, 31/33) [32–34].

The presence of both *Acanthamoeba* and *Vermamoeba* were revealed with high values for both turbidity and TDS. High turbidity showed that the water contained other soluble matters that can provide nutrition and encourage growth rate for the amoeba. The high reading of turbidity demonstrated that these sediments supported the microbial growth and in return, becoming the source of food for the amoeba [35]. This finding also supported by a study conducted in the Philippines’s water sources with the occurrence of *Acanthamoeba* and *Naegleria* that were rich in TDS [19]. This scenario will lead to reduction of water quality which reflects the nutrient availability that could help the parasites to revive in a suitable environment. The result also showed the ability of *A*. *triangularis* to uphold high concentration of nitrite (> 0.5 ppm) and ammonia (> 0.5 ppm) [36]. The high content of both nitrite and ammonia assist in the growth of Gram-negative bacteria that may become the food source for *A*. *triangularis*. In addition, nitrite was also found to be a good indicator for the presence of other parasites, namely, *Cryptosporidium* and *Giardia* [19, 37].

Another study was conducted in France, showing a wide biodiversity of FLA in water treatment plants that used river water as its main source [38]. A similar study was also carried out in Sarawak ofEast Malaysia that revealed the presence of FLA in various processing sites in treatment plants [39]. Relatively, similar treatment processes that include chlorination and filtration are practiced in Myanmar and Laos. Given this, the treated water in both aforementioned countries was contaminated with *V*. *vermiformis*. This may be due to the ability of *V*. *vermiformis* cyst to resist the harsh condition of physical and chemical treatments that are used in the treatment plants.

Cultivation of FLA obtained from water samples was carried out to impede excessive growth of unwanted contaminants such as bacteria and fungi. Hence, this technique was chosen as it is able to grow large quantity of FLA within a short period of time. FLA is able to grow at room temperature (i.e. 25°C), as higher temperature may trigger the overgrowth of bacteria instead of amoeba. The high ratio of bacteria to amoeba (i.e. 10 > 1) that contained in the uncultured water can suppress the growth of *Acanthamoeba* [40]. The cultivation method was performed on the NNA (non-nutrient agar) with non-mucoid bacteria (i.e. *Escherichia coli*) as the food source. In addition, bacteria with mucoid were not preferable as it may delay the phagocytosis by amoeba that may leads to bacterial overgrowth. Sub-cultivation was also performed to assist in removing debris that may contain living/dead bacterial cells, trapped inorganic particles, and organic fibers [41]. Furthermore, the contaminants may obstruct the detection of FLA, especially those obtained from environmental water samples. Staining of the stages of all pathogenic FLA revealed a better observation of the features (i.e. shape) and cellular organelles. The smears of the samples were firstly fixed using methanol to prevent the trophozoites and cysts to become distorted or shrunk. Giemsa, which is commonly used for blood parasites, has produced good contrast against the background for both trophozoites and cysts in the present study. Nevertheless, the space between the endocyst and ectocyst were also stained blue and can cause confusion in determining or confirming the distinct features of both layers. Moreover, trophozoite possessed a better contrast due to its larger size in comparison to cysts. The findings from this study showed that Giemsa stain can be used, especially for routine screening to identify the developmental stages of FLA in general, and trophozoites detection in particular, for clinical epidemiology and public health purposes.

In addition, fluorescence method is used in FLA identification based on its concept of conjugation of protein-antigen and proved to be useful because of the failure of Giemsa stain intake by the cysts. Only endocyst was stained with bright apple-green colour and able to differentiate the number of arms and polygonal shapes for detailed morphological classification of pathogenic *Acanthamoeba*. Hence, it is revealed that the endocyst of FLA consisted of cellulose, a similar material that covers the (oo)cysts of *Cryptosporidium* and *Giardia* [42] that enables simultaneous detection in detecting waterborne parasites containing FLA, *Cryptosporidium*, and *Giardia*.

Overall, observation of the trophozoite stage of FLA by microscopy has permitted the differentiation at genus level. The trophozoites of *Naegleria* were smaller in size as compared to those of *Acanthamoeba* and *Vermamoeba* and were observed to move faster in a unidirectional manner. *Vermamoeba* trophozoites with a transient cylindrical form possessed similar pattern of movement as *Naegleria*. *Acanthamoeba* showed multiple shapes of trophozoites and moving by the projection of lobopodia/acanthopodia in multidirectional. It is known that the classification of FLA based on morphological criteria is insufficient, while their identification is not problematic using different PCR assays, including conventional PCR.

The diagnosis of infection and identification of pathogenic FLA remains unsatisfactory, time-consuming, expensive, laborious, and prone to ethical issues when mouse pathogenicity test was taken into consideration. Due to the advancements in molecular detection, conventional PCR has been developed and seem to be reliable method for routine screening of FLA in environmental samples. One-step based PCR, paired with specific primer by targeting the region of ITS and 18S rDNA [27] is sufficient enough to differentiate between and within species of various organisms. Furthermore, the primer sets used in the present study showed high specificity and capable to amplify a product from potentially pathogenic FLA genotypes, but not with other closely related genera of amoeba. For example, the JDP1-JDP2 primers had been proven to produce an amplicon even from a single trophozoite of *Acanthamoeba* [27].

The internal transcribed spacers (ITS) region has been chosen to study the differences within the genus of *Naegleria* [43]. The ITS analysis has been used in studying heterogeneity in several organisms such as *Cryptosporidium parvum*, trichomonadid protozoa, and *Valkamphia* [42,44]. The species-specific primer was used to detect the presence of pathogenic *N*. *fowleri* [45]. Nevertheless, no positive amplification of *N*. *fowleri* was obtained against the *Naegleria*-like isolates. This might be due to the fact that *N*. *fowleri* is a thermophilic organism that proliferates at an ambient temperature (as high as 45°C) [46].

On the other hand, four of the environmental *Acanthamoeba*-like isolates were successfully amplified using the species-specific primer of JDP1 and JDP2, producing approximately 460 bp amplicons of potentially pathogenic *A*. *triangularis* and *A*. *lenticulata*. The sequence of 18S rDNA from *Acanthamoeba* isolates led into 13 different lineages containing either single or complexes of species. Based on the classification, both *A*. *triangularis* and *A*. *lenticulata* had been classified as type T4 and T5, respectively, and often been associated with human systemic infection and keratitis. Many previous studies similarly reported that T4 is the most prevalent genotype among both clinical specimens and environment samples. The presence of both isolates in our samples probably reflects their better adaptation to various growth conditions relative to isolates from other genotypes.

Morphology of *Vermamoeba vermiformis* isolates from the present study was similar to previously reported species under the microscope [47]. The typical trophozoites of *Vermamoeba* displayed worm-like cell body, stable anterior hyaline cap, and possess a tendency branch when they changed a direction. The 18S rRNA gene sequences of all reported *V*. *vermiformis* isolates in present study showed extremely high similarity with more than 96% identical to AF426157 and KC188996. Further, phylogenetic analysis of 18S rRNA gene sequences in Amoebozoa revealed that Hartmannellidae and Vermamoebidae are paraphyletic and monophyletic group, respectively.

Findings conducted throughout the world had reported that the *Vermamoeba* species is considered pathogenic that is also tolerant towards high temperature [48]. The thermotolerance behaviour is one of the factors that contributed to the virulence characteristic that able to cause diseases and leads to death. Interestingly, our study revealed positive isolate from cold condition of ice cube that are similar to the result carried out in Spain [31]. Thus, it was purported that *V*. *vermiformis* has the ability to survive in various conditions, besides exhibiting thermotolerant properties.

The presence of these parasites is rather alarming due to its ability in causing parasitic infections. Site observation in Myanmar and Laos revealed that fishing activities are prominent in the rivers and ponds. Consumption of these contaminated fishes (raw or under-cooked) with potentially pathogenic FLA could cause a fatal disease to humans. A previous study revealed that freshwater fishes contained several *Hartmannella vermiformis* strains found from its organs [49], thus, there is a possibility that fish can be a host for this parasite. However, FLA transmissions via food are still questionable and more studies in the future need to be carried out to further proven the presence of amoeba within host tissues.

For the past decade, keratitis cases had increased dramatically parallel with the popularity of contact lens usage, hence, resulting in more reports of clinically confirmed cases in Southeast Asia. *Acanthamoeba* species was revealed to be the main cause of keratitis infection in raw water in Thailand [50,51] and Vietnam [52], in regards with water-related activities (i.e. swimming and diving) and accidental water-splashing. In the present study, *A*. *triangulari*s was found in fish pond sample obtained in Myanmar. A previous study has confirmed the role of *A*. *triangularis* in causing human keratitis [53]. Usually, *Acanthamoeba* spp. can be isolated from environmental water that is rich with sediments and other particulate matters [54]. In addition, our study confirmed the presence of *A*. *lenticulata* in water sources such as Mekong River, well water, as well as recreational lake in Myanmar and Laos. This amoeba has also been reported causing granulomatous amoebic encephalitis (GAE) in immunocompromised patients [55].

The occurrence of FLA in treated water cannot be neglected due to the increasing reports of keratitis in Malaysia [56], Indonesia [57], and the Philippines [58]. Keratitis had always been associated with the infection caused by *Acanthamoeba*, but *Hartmannella* (= *Vermamoeba*) too, is able to cause similar symptoms of watery eyes, redness and blurred vision [59,60]. In the present study, *V*. *vermiformis* was isolated mostly from treated water of swimming pools from Myanmar and Laos. This situation revealed that *V*. *vermiformis* is able to adapt in chlorinated water and showed the ability to survive in the ecology. Although limited epidemiological data available, chlorinated swimming pool can possibly be a new niche for FLA to survive. The alarming phenomenon is considered a major concern, especially to contact lens wearers as they are susceptible to keratitis infection if lens are not appropriately handled especially before and after entering the pool. In addition, the pipe that connects water into the swimming pools may contain biofilm that support the colonization of FLA [61].

From this finding, the isolation of FLA revealed a better understanding on the distribution and prevalence of both pathogenic *Acanthamoeba* and *Vermamoeba* in Myanmar and Laos. The high occurrence of amoebae in Myanmar must not be neglected by the local authorities to ensure good water quality is supplied to the consumers. Proper precaution measures in ensuring water quality must be carried out to assist in regular monitoring on any possibilities of the emerging amoebae. This is because of the wide occurrences of FLA is not only limited to one species at any particular water source or area. Meanwhile, water that is commonly used by public, such as swimming pool, must undergo efficient and appropriate treatment processes. The usage of alternative disinfectants such as Baquacil, chlorinated cyanurate and chlorine dioxide can be added into the water to control the growth of FLA [62,63]. Raw water that is used for recreational purposes (i.e. hotspring, coastal) must also be monitored closely as the untreated water may contain high number of FLA, thus, can transmit infections to the public [64,65]. In addition, the infection caused by FLA is still misdiagnosed or under-reported, particularly in this region. Thus, future investigations of the occurrence as well as distribution of FLA in treated and untreated water with a larger sample size need to be taken in serious consideration. The findings from the investigations will be reported to relevant authorities to prevent further widespread of water contamination with potentially pathogenic species of these free-living parasites.
