# Correction: Pathogenic waterborne free-living amoebae: An update from selected Southeast Asian countries

**DOI:** 10.1371/journal.pone.0177564

**Published:** 2017-05-08

**Authors:** Mohamad Azlan Abdul Majid, Tooba Mahboob, Brandon G. J. Mong, Narong Jaturas, Reena Leeba Richard, Tan Tian-Chye, Anusorn Phimphila, Panomphanh Mahaphonh, Kyaw Nyein Aye, Wai Lynn Aung, Joon Chuah, Alan D. Ziegler, Atipat Yasiri, Nongyao Sawangjaroen, Yvonne A. L. Lim, Veeranoot Nissapatorn

The affiliation of the 14^th^ author is incorrect. The correct affiliation is: Department of Microbiology, Faculty of Science, Prince of Songkla University, Hat Yai, Thailand.
